# Bmi-1 induces radioresistance by suppressing senescence in human U87 glioma cells

**DOI:** 10.3892/ol.2014.2606

**Published:** 2014-10-10

**Authors:** LAN YE, CUIHONG WANG, GUANYING YU, YUHUA JIANG, DIANSHUI SUN, ZAIYUN ZHANG, XIAOMING YU, XIAOMEI LI, WEI WEI, PING LIU, JIAN CHENG, BIN DU, LIKUAN HU

**Affiliations:** 1 Cancer Center, The Second Hospital of Shandong University, Jinan, Shandong 250033, P.R. China; 2 Jinan Central Hospital, Jinan, Shandong 250014, P.R. China; 3 Department of Radiotherapy, Qilu Hospital, Shandong University, Jinan, Shandong 250012, P.R. China

**Keywords:** glioma, Bmi-1, senescence, radiation

## Abstract

Radiotherapy is the main locoregional control modality for a number of types of malignant tumors, including glioblastoma. However, radiotherapy fails to prevent recurrence in numerous patients due to the intrinsic radioresistance of cancer cells. Cell senescence is significant in tumor suppressor mechanisms and is closely associated with the radioresistance of cancer cells. Bmi-1 has been proposed to be an oncogene that can induce anti-senescence in tumor cells. The present study investigated the response of U87 glioma cells to radiation exposure and the role of Bmi-1 in the response following radiotherapy. Cell apoptosis and cell cycle distribution were assessed using flow cytometry, and a SA-β-Gal stain was used to observe the senescence ratio of U87 cells following radiation. The expression of Bmi-1 in U87 cells exposed to different doses of radiation was evaluated by western blot analysis. X-ray radiation was found to inhibit U87 cell proliferation through the induction of senescence rather than apoptosis. Following exposure to radiation, the cell cycle distribution was dysregulated, with an increased number of cells in the G_2_/M phase, and the expression of Bmi-1 was upregulated, particularly when a dose of ≥6 Gy was administered. The results indicated that senescence is the main mechanism by which U87 cell growth is inhibited following radiation. In addition, Bmi-1 may be significant in increasing the radioresistance of glioma cells by enabling cell senescence.

## Introduction

Gliomas are the most common type of brain tumor, worldwide (1). Despite the use of surgery, radiation and conventional chemotherapy, the median survival time of patients with the most malignant type of glioma, glioblastoma, is only one year (2). Gliomas may be characterized by resistance to apoptosis and radiation (3,4), however, the mechanism of this process remains unclear.

According to previous studies, certain types of glioma cells, including U87 cells, undergo senescence as opposed to apoptosis following exposure to ionizing radiation (5–7). Cellular senescence is an extremely stable form of cell cycle arrest and constitutes a strong natural tumor suppressor mechanism. At present*,* the predominant senescence signaling pathways include the p53-p21Cip1/Waf1, p19Arf-Mdm2 and p16Ink4a-Rb pathways (8).

B lymphoma Mo-MLV insertion region 1 homolog (Bmi-1) is a polycomb group protein that regulates cell proliferation and has been found to be upregulated in a variety of human cancer types, including acute myeloid leukemia and breast, colon, lung, ovarian and nasopharyngeal cancer; this suggests a potential role of Bmi-1 as an oncogene (9–12). Bmi-1 has been demonstrated to regulate the expression of the p16Ink4a, hTERT and Hox group genes (13). Overexpression of Bmi-1 may reduce the expression of p16 and p19Arf (14,15), which induce anti-senescence in tumor cells. Bmi-1 has also been reported to be overexpressed in certain gliomas that usually possess a poor prognosis (16–18).

The current study investigated the response of U87 glioma cells to radiation exposure as well as the role of Bmi-1 in their response following radiotherapy.

## Materials and methods

### Cell culture

The human glioma cell line U87 was purchased from the Cell Bank of the Chinese Academy of Sciences (Shanghai, China). The glioma cells were maintained in minimum essential medium (GE Healthcare Life Sciences, Logan, UT, USA) containing 10% fetal bovine serum (FBS; GE Healthcare Life Sciences) and incubated at 37°C in a humidified atmosphere of 5% CO_2_.

### Radiation

The cells were plated on 24-well dishes at a density of 5×10^4^ cells/0.5 ml on day 0. Subsequently, the U87 cells were immediately exposed to X-ray radiation with a linear accelerator source (Elekta, Stockholm, Sweden) at a dose rate of 300 cGy/min. Every 24 h, the number of cells in three wells was quantified using a cell counter (Inno-Alliance Biotech, Wilmington, DE, USA) and the mean was calculated. The results are presented as the mean ± standard error of three independent experiments.

### Annexin V-fluorescein isothiocyanate (FITC)/propidium iodide (/PI) double-labeled flow cytometry (FCM)

To detect the apoptotic ratio of cells following 72 h of exposure to X-ray radiation, the expression of Annexin V-FITC (Sigma-Aldrich, St. Louis, MO, USA) and the exclusion of PI (Sigma-Aldrich) were detected by two-color FCM using the LSR Fortessa cell analyzer (Becton-Dickinson, Franklin Lakes, NJ, USA). The U87 cells were collected in Eppendorf PCR tubes, washed twice with phosphate buffered saline (PBS) and resuspended in 500 μl binding buffer. The samples were incubated with 5 μl Annexin V-FITC at room temperature for 10 min and then 5 μl PI was added. Each sample was incubated in the dark for a further 10 min at room temperature prior to the fluorescence intensity being quantitated by FCM.

### Cell cycle

To detect the cell cycle of cells following 72 h of exposure to radiation, glioma cells were fixed with 70% ethanol, resuspended in PBS/1% FBS, and treated with ribonuclease (Beyotime Institute of Biotechnology, Haimen, China). PI was added to the cells and the samples were then analyzed by FCM (Becton-Dickinson). Cell cycle profile analysis of the DNA histograms of integrated red fluorescence was performed with ModFit LT 2.0 (Verity Software, Inc., Topsham, ME, USA).

### Senescence-associated β-galactosidase (SA-β-Gal) staining

To detect the senescence ratio of cells following 72 h of exposure to radiation, SA-β-Gal staining was performed using the SA-β-Gal Kit (Beyotime Institute of Biotechnology) following the manufacturer’s instructions. The cells were considered to be positive when the cytoplasm was stained with SA-β-Gal.

### Western blot analysis

The U87 cells were collected and lysed in lysis buffer (150 mM NaCl, 50 mM Tris with pH 7.4, 1% NP40, 0.1% SDS, 0.5% sodium deoxycholate), supplemented with protease inhibitors (CWBio, Inc., Beijing, China) subsequent to 72 h of exposure to radiation, which was followed by centrifugation at 10,000 × g for 15 min at 4°C. The protein concentration in each sample extract was detected using the bicinchoninic acid assay (CWBio, Inc.). SDS-PAGE was performed on 15% polyacrylamide gels, with 40 μg of protein sample per lane. Following electrophoresis, the protein was transferred to nitrocellulose membranes and incubated in 5% non-fat milk at room temperature for 2 h. Subsequently, the membranes were incubated overnight at 4°C with a primary polyclonal rabbit anti-human antibody for Bmi-1 (sc-10745; Santa Cruz Biotechnology, Dallas, TX, USA). Subsequent to being washed three times using PBS, the membrane was incubated with an appropriate concentration of horseradish peroxidase-conjugated anti-rabbit secondary antibody (CWBio, Inc.) for 2 h. Following a further three washes with PBS, the specific protein band was visualized using an enhanced chemiluminescence kit (CWBio, Inc.).

### Statistical analysis

Each experiment was conducted in triplicate. Student’s T-test was used to evaluate the statistical significance of the differences and a P<0.05 was considered to indicate a statistically significant difference.

## Results

### Proliferation inhibition in U87 glioma cells following X-ray radiation exposure

**Figure 1:**
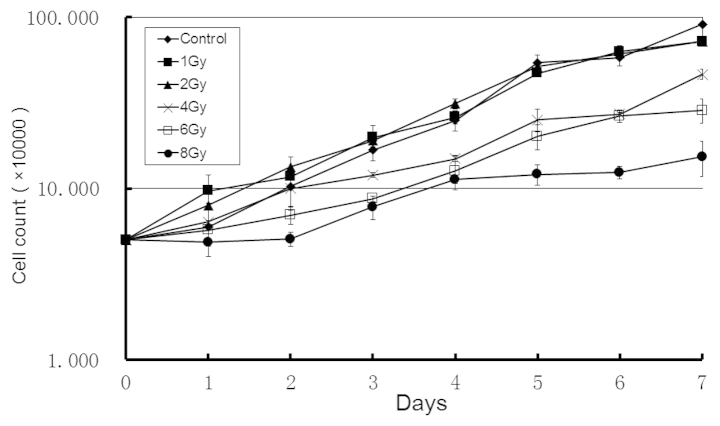
Cell growth curves of U87 glioma cells following exposure to X-ray radiation. The cells were plated on 24-well dishes at a density of 5×10^4^ cells/0.5 ml on day 0 and immediately exposed to X-ray radiation at various doses. Every 24 h, the number of cells was quantified using a cell counter. The results are presented as the mean ± standard error of three independent experiments.

To determine the cellular proliferation of U87 cells following exposure to radiation, the U87 cells were treated with X-ray radiation at 1, 2, 4.6 and 8 Gy (Fig. 1). Cellular proliferation was observed to be inhibited in a dose-dependent manner at doses ≥4 Gy. No significant inhibition of cellular proliferation was observed in the control, 1 and 2 Gy groups. Following 24 h of exposure to X-ray radiation, the number of cells in the groups receiving doses of 1 and 2 Gy X-ray radiation was increased compared with the control groups (P<0.05).

### Apoptosis and senescence in U87 glioma cells following exposure to X-ray radiation

**Figure 2:**
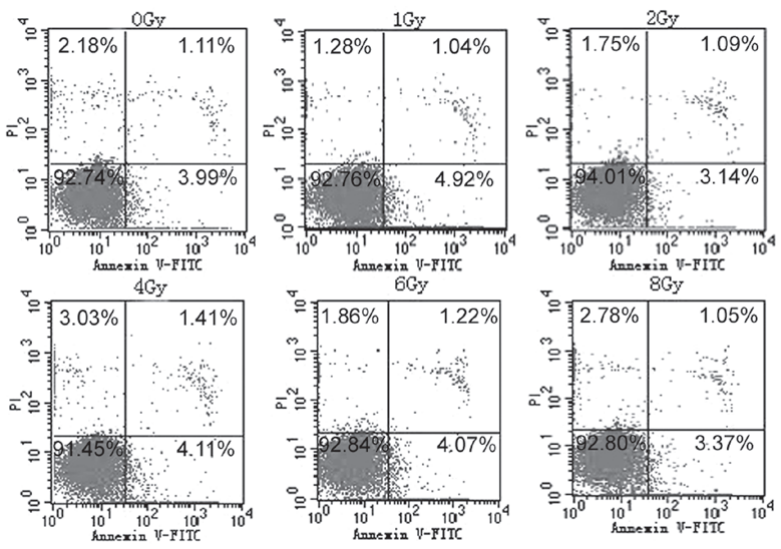
Cell apoptosis. Following 72 h of exposure to X-ray radiation at various doses, the U87 glioma cells were assessed by Annexin V-fluorescein isothiocyanate/propidium iodide double staining.

**Figure 3:**
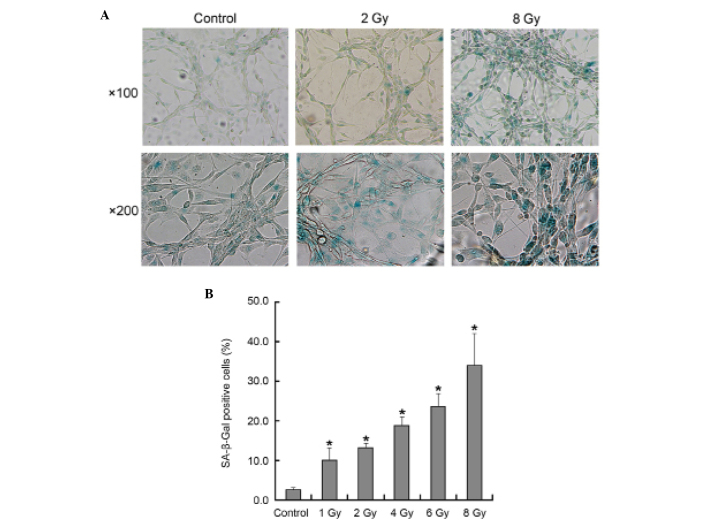
Cell senescence. (A) Following 72 h of exposure to X-ray radiation at various doses, the U87 glioma cells were treated by SA-β-Gal staining and images were captured under phase-contrast microscopy. (B) Under the microscope the SA-β-Gal positive cells were counted and the quantitative results are presented as the mean ± standard deviation of three independent experiments. ^*^P<0.05 vs. control.

To evaluate whether the cellular proliferation inhibition of U87 cells in response to X-ray radiation is associated with apoptosis or senescence, U87 cells were treated with X-ray radiation at 1, 2, 4.6 and 8 Gy. Following 72 h of exposure to radiation, no significant apoptosis was identified in any of the groups (Fig. 2). Using SA-β-Gal staining, it was observed that senescence had occurred in all treatment groups in a dose-dependent manner (Fig. 3B). Under phase-contrast microscopy, the senescent morphology of a large and flattened shape was observed (Fig. 3A). These morphological changes were more evident in the cells that were exposed to radiation doses of ≥6 Gy.

### The change of cell cycle phase in U87 glioma cells following exposure to X-ray radiation

**Figure 4:**
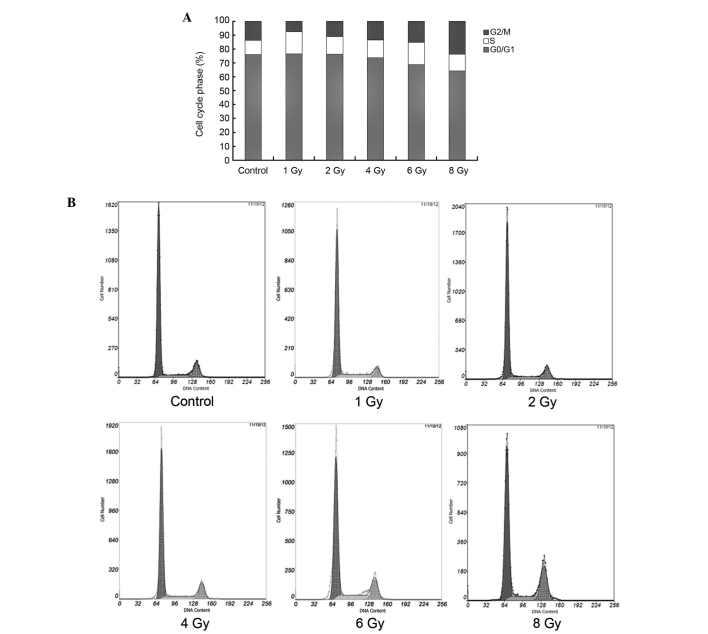
Cell cycle. (A) Following 72 h of exposure to X-ray radiation at various doses, the U87 glioma cells were assessed by propidium iodide staining and analyzed to generate a cell cycle profile. (B) The columns provide the mean of three independent experiments. ^*^P<0.05 vs. control.

Following 72 h of exposure to X-ray radiation, the cell cycle phases of U87 cells were analyzed (Fig. 4). The proportion of cells in the S phase was increased in all of the treatment groups (P<0.05). The proportion of cells in the G_2_/M phase was decreased in the 1 and 2 Gy groups and increased in the 6 and 8 Gy groups (P<0.05). No significant difference was identified in the G_0_/G_1_ phase between the 1, 2, 4 Gy and control groups, however, this was increased in the 6 and 8 Gy groups.

### Expression of Bmi-1 in U87 glioma cells following exposure to X-ray radiation

**Figure 5:**
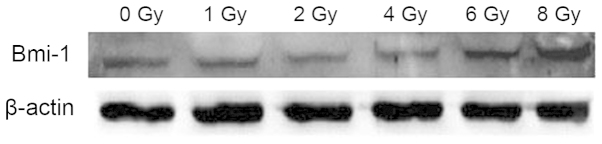
Western blot analysis demonstrating that ionizing radiation increased Bmi-1 expression. β-actin was used as a loading control.

To observe whether the expression of Bmi-1 was associated with exposure to X-ray radiation, U87 cells were exposed to X-ray radiation in doses of 1, 2, 4.6 and 8 Gy. Following 72 h of exposure to radiation, the expression of Bmi-1 was determined (Fig. 5). Bmi-1 expression was increased in the 6 Gy and 8 Gy groups (P<0.05). No significant difference in Bmi-1 expression was observed between the 1, 2 and 4 Gy and control groups.

## Discussion

Glioma is the most common malignant brain tumor in adults, worldwide (19), with grade IV glioblastoma being the most fatal (20,21). Radiation is an important therapy for glioma patients, however, the resistance of gliomas to radiation has affected the response to treatment (22). If the mechanism of radiation resistance is identified, novel treatment options for radiation-resistant gliomas may be investigated to improve the response to radiation treatment. U87 cells were selected for investigation in the current study as they are a human glioblastoma cell line that is characterized by resistance to apoptosis and radiation.

Radiotherapy is significant in the treatment of glioma. Numerous studies have focused on the more effective method of increasing the radiosensitivity of glioma (23–25). Radiation causes DNA damage to cells and subsequently induces apoptosis, senescence or death. Apoptosis is known to be an important mechanism for radiotherapy and chemotherapy, however, the present study and others have demonstrated that radiation did not induce apoptosis in certain gliomas, but instead induced senescence (5,26,27).

Cellular senescence is characterized by irreversible cell cycle arrest, with morphological changes that result in a large and flattened cell shape. Senescent cells remain metabolically active and exhibit certain properties, including SA-β-Gal staining based on lysosomal β-galactosidase activity at pH 6.0, resistance to apoptosis and altered gene expression (28). Tumor cells have been hypothesized to possess the ability to elude senescence. Therefore, it is possible that improved tumor treatments may be developed by understanding the underlying mechanism of senescence (29). The importance of cellular senescence as a tumor suppression mechanism is increasingly being recognized.

Bmi-1 may be significant in the senescence of tumor cells (30). Bmi-1 negatively controls the expression of the p16 gene (31,32); p16 expression is one of the hallmarks of cellular senescence (33) and inhibits the activity of CDK4, which is the key enzyme in the G_1_-S transformation during cell division, preventing the cells from entering S phase. Once p16 is mutationally or transcriptionally inactivated in cells, CDK4 is uncontrolled, leading to malignant proliferation. The tumor suppressor gene, p16, is often mutationally and transcriptionally inactivated in gliomas. In addition, Bmi-1 can induce the coordination of the c-myc gene with p16 to promote cell transformation and tumor formation, which results in the cell escaping apoptosis (34). For these reasons, Bmi-1 presents an attractive target for glioma therapy.

The aim of the present study was to elucidate whether Bmi-1 expression was associated with the senescence of the U87 glioma cells that have been exposed to X-ray radiation. X-ray radiation was unable to induce apoptosis in U87 glioma cells. However, X-ray radiation was able to induce senescence, resulting in the inhibition of U87 cell proliferation following exposure to the radiation at a dose of ≥4 Gy. An increase in the proportion of cells in the S phase was observed following exposure to various doses of X-ray radiation. In addition, in the groups exposed to the lower doses of 1,2 and 4 Gy, the proportion of cells in the G_2_/M phase was decreased or not significantly different compared with the control group. Furthermore, the proportion of cells in the G_0_/G_1_ phase was not significantly different compared with the control group. When cell senescence is induced, the cell cycle should arrest in G_1_ phase. This may be the reason that the proportion of cells in the G_0_/G_1_ phase remained stable despite the increase in the proportion of cells in the S phase. However, in the higher dose group, exposed to 6 and 8 Gy, the proportion of cells in the G_2_/M phase was increased and the proportion of cells in the G_0_/G_1_ phase was markedly decreased. Although the proportion of cells that were SA-β-Gal positive was greater when compared with the control and other radiation groups, which means that an increased number of U87 cells underwent senescence, the U87 cells did not arrest in the G_1_ phase and continued to the S and G_2_/M phases. It appeared that certain factors aid U87 cells in evading senescence following exposure to higher doses of X-ray radiation. In the present study, Bmi-1 expression was observed to be significantly increased in the higher dose (6 and 8 Gy) groups and therefore, may exert an effect that aids the cells in evading cell cycle arrest. However, the mechanism associated with this progress is not fully understood.

In the present study, senescence was determined as the main mechanism of U87 cell growth suppression following exposure to X-ray radiation. Bmi-1 may be significant in the radioresistance of U87 cells by suppressing senescence. Future studies focusing on the expression of Bmi-1 and its downstream genes, using gene transfection technology in additional glioma cell lines, may aid in determining the possible mechanism of Bmi-1 in the radioresistance of glioma cells.
